# Taguchi Optimization of Roundness and Concentricity of a Plastic Injection Molded Barrel of a Telecentric Lens

**DOI:** 10.3390/polym13193419

**Published:** 2021-10-05

**Authors:** Chao-Ming Lin, Yun-Ju Chen

**Affiliations:** Department of Mechanical and Energy Engineering, National Chiayi University, Chiayi 600355, Taiwan; yunju.chen.zoe@gmail.com

**Keywords:** plastic optical barrel, injection molding, roundness, concentricity, Taguchi method

## Abstract

Plastic is an attractive material for the fabrication of tubular optical instruments due to its light weight, high strength, and ease of processing. However, for plastic components fabricated using the injection molding technique, roundness and concentricity remain an important concern. For example, in the case of a telecentric lens, concentricity errors of the lens barrel result in optical aberrations due to the deviation of the light path, while roundness errors cause radial stress due to the mismatch of the lens geometry during assembly. Accordingly, the present study applies the Taguchi design methodology to determine the optimal injection molding parameters which simultaneously minimize both the overall roundness and the overall concentricity of the optical barrel. The results show that the geometrical errors of the optical barrel are determined mainly by the melt temperature, the packing pressure, and the cooling time. The results also show that the optimal processing parameters reduce the average volume shrinkage rate (from 4.409% to 3.465%) and the average deformations from (0.592 mm to 0.469 mm) of the optical barrel, and the corresponding standard deviation values are reduced from 1.528% to 1.297% and from 0.263 mm to 0.211 mm, respectively. In addition, the overall roundness and overall concentricity of the barrel in the four planes are positively correlated.

## 1. Introduction

Plastic injection molding is a fast and economical process for fabricating optical components with high precision, excellent performance, and good strength-to-weight properties. However, the quality of injection molded parts is highly dependent on the choice of processing parameters, including the melt temperature, mold temperature, filling time, packing time, packing pressure, injection pressure, and so on. As a result, a proper control of the processing conditions is essential [1,2,3,4,5,6,7].

**Figure 1:**
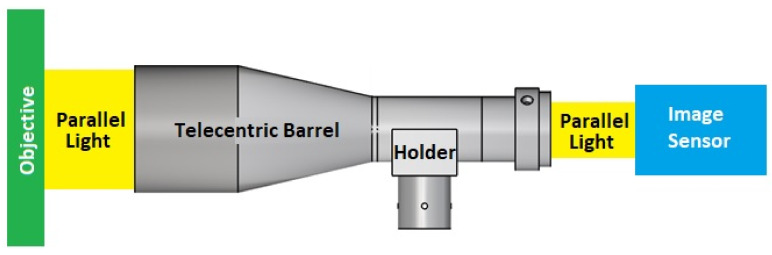
Coaxial bilateral telecentric optical system.

Compared to conventional lenses, in which the magnification varies with the distance between the lens and the object, telecentric lenses have a constant field of view at all distances from the lens. As a result, they eliminate the parallax error inherent in traditional fixed-focal length lenses and, therefore, find widespread use in machine vision-based systems where precise and repeatable measurements are required, such as metrology, microlithography, semiconductor manufacturing, and so on [8,9,10,11,12]. Figure 1 presents a simple schematic illustration of a typical coaxial bilateral telecentric optical system consisting of a telecentric barrel, a coaxial light source, a holder, and an optical imaging system.

Among these components, the telecentric barrel plays a critical role in ensuring that the light emitted by the coaxial light source follows the preset path. In particular, the roundness and concentricity of the barrel must be strictly controlled in order to ensure a proper placement and alignment of the internal lens [13,14,15,16,17,18]. The lens barrel is generally fabricated from plastic material due to the latter’s light weight and ease of manufacturing. However, as shown in Figure 1, the barrel has a varying tube diameter and an asymmetric geometry (due to the presence of the holder). Thus, controlling the injection molding parameters in such a way as to minimize the roundness and concentricity errors caused by the shrinkage and deformation of the barrel during the molding process represents a significant challenge [19,20].

In general, the Taguchi method and mold flow analysis provide a convenient and cost-effective approach for optimizing the processing conditions employed in the injection molding process. Accordingly, the present study employs a hybrid approach consisting of the Taguchi design method and mold flow simulations to determine the optimal settings of the main injection molding parameters (i.e., the injection pressure, the packing pressure, the melt temperature, the mold temperature, and the cooling time) for simultaneously minimizing both the roundness and the concentricity errors of the telecentric barrel. Having determined the optimal processing conditions, a further investigation is performed to examine the correlation between the overall roundness and the overall concentricity of the barrel and the effects of the optimal processing conditions on the average volume shrinkage rate of the barrel following removal from the mold [21,22,23,24,25].

## 2. Theoretical Analysis

In the present study, the mold flow analysis is performed using Moldex3D computer aided engineering (CAE) simulations. The simulations assume a contact interface between the part’s surface and the mold wall and separate the warpage analysis process into two parts, namely, in-mold deformation during the packing and cooling stages and free deformation following ejection from the mold. For the geometric accuracy requirements of the coaxial bilateral telecentric barrel, the final deformation analysis of the cured part from the temperature after demolding to the room temperature will be calculated based on the analysis method of the roundness and concentricity. The related theoretical calculations are explained as follows.

### 2.1. Flow Analysis during Filling Stage

Using the Moldex3D solid simulation, the polymer melt flow develops during the filling stage of the injection molding process where the melt flow is assumed to be incompressible. The polymer melt is assumed to be Generalized Newtonian Fluid (GNF). Therefore, the non-isothermal 3D flow motion can be mathematically described by the mass, momentum conservation, and energy conservation equations, which can be written as follows [26]:

(1) $$\frac{\partial \rho }{\partial t}+\nabla \cdot \rho u=0$$

(2) $$\frac{\partial \left(\rho u\right)}{\partial t}+\nabla \cdot \left(\rho uu-\sigma \right)=\rho g$$

(3) $$\rho c_{p}\left(\frac{\partial T}{\partial t}+u\cdot \nabla T\right)=\nabla \cdot \left(k\nabla T\right)+\eta {\dot{\gamma }}^{2}$$

where *u* is velocity, *p* is the pressure, *ρ* is the density, *c_p_* is the heat capacity, *η* is the viscosity, $\dot{\gamma }$ is the shear rate, *k* is the heat conductivity, and *σ* is the stress tensor; this can be expressed as follows:

(4) $$\sigma =-pI+\eta \left(\nabla u+\nabla u^{T}\right)$$

Considering the constitutive equation for the general polymer materials, the Modified-Cross viscosity [27] model with Arrhenius temperature is used to describe the rheological property of the polymer melt.

(5) $$\eta \left(T,\dot{\gamma }\right)=\frac{\eta_{0}\left(T\right)}{1+{\left(\eta_{0}\dot{\left(T\right)\gamma }/\tau^{*}\right)}^{1-n}}$$

(6) $$\eta_{0}\left(T\right)=Be^{\left(\frac{T_{b}}{T}\right)}.$$

where η is the viscosity, $\eta_{0}$ is the melt viscosity under zero-shear-rate conditions, $\tau^{*}$ is the parameter that describes the transition region between zero shear rate and the power law region of the viscosity curve, *n* is the Power Law index, and B is the consistency index. The Modified-Cross viscosity model includes the Newton’s fluid interval and the power-law shear thinning interval. When the shear rate approaches zero, this model predicts the zero-shear rate viscosity $\eta_{0}$; when the shear rate is large, it predicts the power-law behavior. The $\tau^{*}$ in this model is a constant, which physically represents the critical shear stress value for the transition from Newtonian fluid to power law fluid. Compared with the other model, the Modified-Cross model requires fewer parameters and can capture the dependence of viscosity on the shear rate. Therefore, the Modified-Cross model is often used in commercial simulation software, just like the Moldex3D software in this study.

A volume fraction function, *f*, is introduced to track the advance of the melt front. Here, *f* = 0 is defined as the air phase and *f* = 1 as the polymer melt phase. Hence, the melt front is located within cells with 0 < *f* < 1. The advancement of f over time can be expressed as the following transport equation:

(7) $$\frac{\partial f}{\partial t}+\nabla \cdot \left(uf\right)=0$$

After the part is ejected from the mold, a free thermal shrinkage happens due to the temperature and pressure difference. The warpage analysis assumes the mechanical properties are elastic. The stress–strain equilibrium equations enable us to solve the problems.

### 2.2. Shrinkage and Warpage

The temperature and pressure changes which occur during the injection molding process result in corresponding changes in the specific volume and density of the polymer. These changes lead in turn to a warpage of the molded component as it cools from the melt condition to the solid condition. The part additionally undergoes volume shrinkage during the molding process and following its removal from the mold. During the packing stage, the shrinkage reduces as the packing pressure and packing time increase.

For semi-crystalline polymers, the shrinkage behavior mainly depends on the degree of crystallization. If the mold temperature is low and the cooling rate is high, it is not easy to crystallize, but there is a small shrinkage; on the other hand, if the mold temperature is high and the cooling rate is low, the macromolecular chain has enough relaxation time and easily to form crystals. The amount of shrinkage will naturally increase.

For isotropic materials, the linear shrinkage is one-third the volumetric shrinkage (see Equation (8) below). However, in the injection molding process, the orientation effect of the polymer forming and the shrinkage behavior are both constrained by the mold wall. As a result, the shrinkage deformation exhibits a non-isotropic behavior and the linear shrinkage in the part thickness is thus governed by Equation (9) [28,29,30,31,32,33].

(8) $$S_{L}=1-{\left(1-S_{V}\right)}^{1/3}\approx \left(\frac{1}{3}\right)S_{V}$$

(9) $$S_{L}\approx 0.9-0.95S_{V}$$

where *S_L_* is the linear shrinkage rate, and *S_V_* is the volume shrinkage rate.

### 2.3. Stress Analysis after the Demolding Stage

In this stage, the plastic forming part is no longer restricted to the mold after demolding and is in the free shrinking stage. The free volume shrinkage of the molded component following its removal from the mold depends mainly on the thermal stress induced by the difference between the temperature of the part and that of the environment. If the shrinkage stress exceeds the mechanical strength of the part, the part undergoes warpage. Conversely, if the plastic part is sufficiently strong to resist the thermal stress, the part retains its original geometry and dimensions. However, shrinkage voids may still be formed within the plastic component, which degrade the mechanical properties of the part and may lead to cracks and breakage under the effects of an external force. In the warpage analysis of the Moldex3D solid model, the assumptions are as follows: the material property is linear and elastic; there is a small amount of strain; the behavior is approximately steady; and the plastic part is elastically deformed. The governing equations for the material behavior in the warpage analysis can thus be expressed as follows [34]:

(10) $$\sigma_{ij,j}+f_{i}=0$$

(11) $$\sigma_{ij}=C_{ijkl}\left(\varepsilon_{kl}-\varepsilon_{kl}^{0}-\alpha_{kl}\cdot \Delta T\right)+\sigma_{ij}^{F}$$

(12) $$\varepsilon_{ij}=\left(u_{i,j}+u_{j,i}\right)/2$$

where $\sigma_{ij}$ is the stress tensor, $\sigma_{ij}^{F}$ is the initial stress induced by the flow, $\varepsilon_{ij}$ is the infinitesimal elastic strain, $\varepsilon_{ij}^{0}$ is the initial strain from the P-*v*-T relationship, $C_{ijkl}$ is the elastic material stiffness, $\alpha_{kl}$ is the coefficient of linear thermal expansion, and ΔT is the temperature difference.

### 2.4. Roundness Evaluation

**Figure 2:**
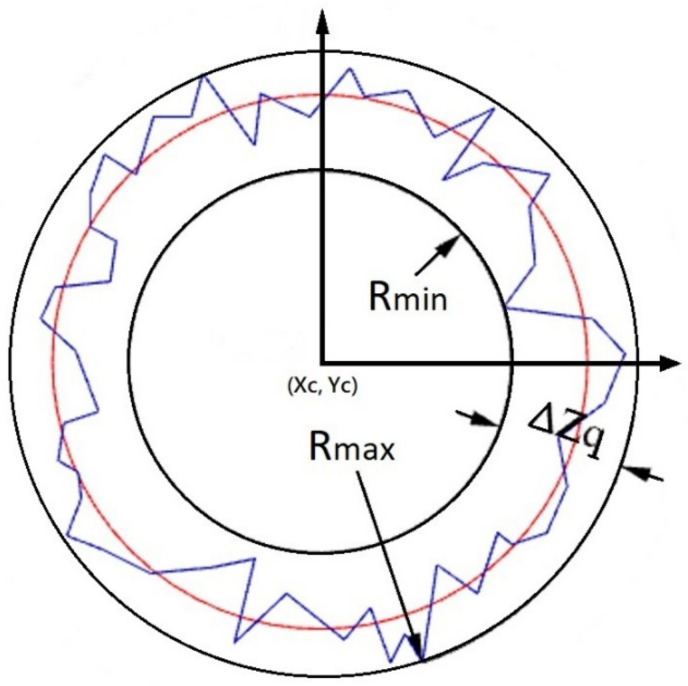
Least squares circle method for determination of roundness (Δ*Zq*) at specified Z-plane.

The most common methods for determining roundness errors include the Least Squares Circle (LSC) method, the Minimum Zone Tolerance Circle (MZC) method, the Maximum Inscribed Circle (MIC) method, and the Minimum Circumscribed Circle (MCC) method [35]. Figure 2 illustrates the LSC method, in which the center of the circle is first determined by identifying the circular contour which minimizes the sum of squared error (SSE) between the inner and outer radii of the interior surface (shown in red in Figure 2). This center point is then used to draw the circumscribed and inscribed circles of the barrel interior surface, respectively (see two black lines in Figure 2). Finally, the roundness of the circle (Δ*Zq*) is quantified as the radial distance (*R_max_*–*R_min_*) between them [36]:

(13) $$\mathrm{Roundness}=\Delta Zq=R_{max}-R_{min}$$

In practice, the center point of the least square circle is unique, and its accuracy depends on the number of measurement points [37,38,39,40]. The overall roundness including n planes can be defined as

(14) $${\mathrm{Roundness}}_{\mathrm{overall}}=\overset{n}{\underset{i=1}{\sum }}{\left[{\left(\Delta Zq\right)}_{i}\right]}^{2}/n$$

After the above calculation, one can get the center of each contour (*Xc, Yc*), the roundness of each contour, and the overall roundness of all contours after the injection molding processing.

### 2.5. Concentricity Evaluation

**Figure 3:**
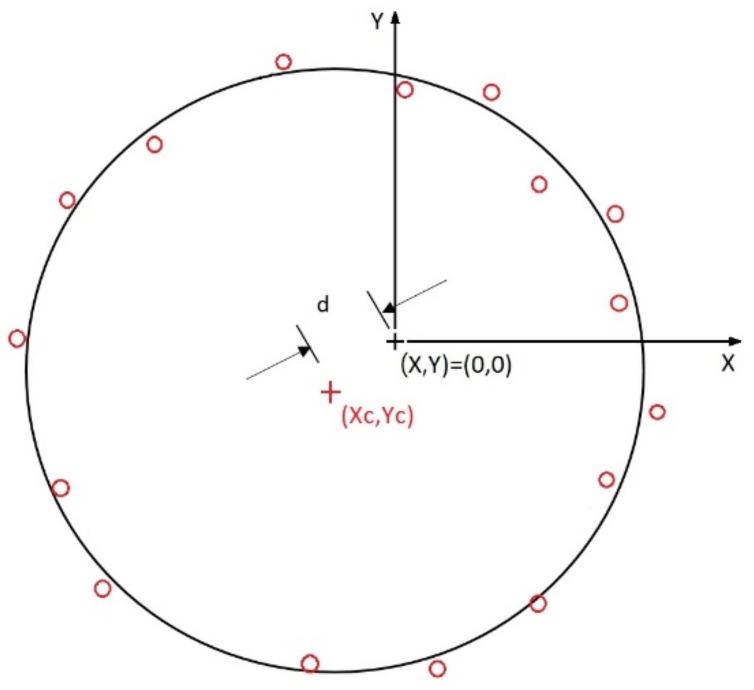
Calculation of concentricity (d) at specified Z-plane.

Concentricity refers to the deviation of the center of a circle or center of a cylinder from the center of the reference form. It is generally evaluated as either the axis concentricity tolerance (with the tolerance zone centered on the axis of the reference form) or the point concentricity tolerance (with the reference point taken as the center of the circle). For either method, the concentricity measurement process involves establishing the coordinates of the required checking plane, measuring the position of the center of the contour circle that needs to be compared after setting the datum, and calculating the distance between the original center of the circle and the center of the actual contour. As with the roundness evaluation described above, the accuracy of the concentricity tolerance process also increases with an increasing number of measurement points. Figure 3 illustrates the concentricity evaluation process for the case where the reference coordinates (*X,Y*) are set as (0,0) and the coordinates of the fitted circular contour are denoted as (*Xc, Yc*). The concentricity *d* at specified Z-plane is then evaluated simply as [41].

(15) $$\mathrm{Concentricity}=d=\sqrt{{\left(X_{c}-X\right)}^{2}+{\left(Y_{C}-Y\right)}^{2}}$$

The overall concentricity including n contours can be defined as

(16) $${\mathrm{Concentricity}}_{\mathrm{Overall}}=\sum_{i=1}^{n}{\left[d_{i}\right]}^{2}/n$$

## 3. Methods and Procedures

The geometry model of the telecentric lens barrel and positions of the tracked nodes during the simulations were defined in **Rhinoceros**. The model was then imported into **Moldex3D** to design the mold cavity and the gate, runner and cooling system, as well as to perform the molding flow simulations. The simulations considered the use of **PA66** polymer material with the properties shown in Table 1 as the feedstock material. The total warpage was obtained directly from the output results of the mold flow analysis, while the roundness was calculated based on the distance between each tracked node and the offset center, and the concentricity was computed as the shortest distance between the offset circle center and the original axis.

### 3.1. Material Characteristics and P-v-T curves of PA66

**Figure 4:**
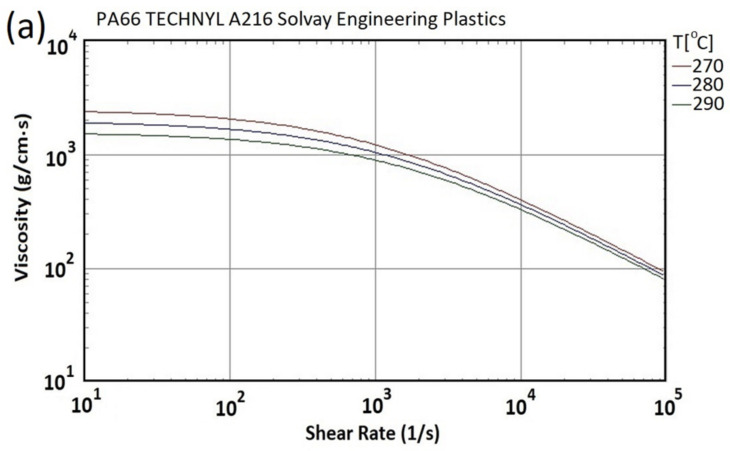

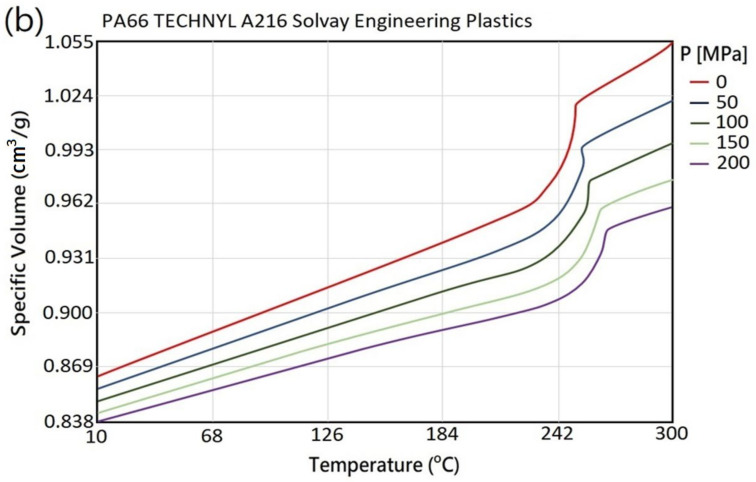
(**a**) Viscosity and (**b**) P-*v*-T properties of PA66 material (Source: Moldex3D material library).

Figure 4a shows the relationship between the viscosity of PA66 and the shear rate at different temperatures as the basis for subsequent mold flow analysis, and the Modified-Cross viscosity [27] model (See Equations (5) and (6)) with Arrhenius temperature is used to describe the rheological property of the polymer melt. Figure 4b shows the P-*v*-T relationship diagram of PA66. In the process of plastic processing, the plastic undergoes a very rapid cooling process under the temperature and pressure controlled by the molding process and changes from a molten state to a solid state. Usually, the volume changes greatly, and a simple comparison is no longer possible. To describe the capacity constant, the relationship between specific volume/pressure/temperature characteristics (P-*v*-T) is determined to calculate the degree of compression of the material in the packing stage, as well as the shrinkage rate and shrinkage warpage of the final plastic part after ejection.

The Modified Tait Model II [26] is used to describe the P-*v*-T relationship of semi-crystalline materials (PA66) and is also the recommended P-*v*-T model in Moldex3D.

(17) $$v\left(T,P\right)=v_{0}\left(T\right)\left[1-Cln\left(1+\frac{P}{B\left(T\right)}\right)\right]+v_{t}\left(T,P\right)$$

where $v\left(T,P\right)$ is the specific volume; $v_{0}$ is the specific volume at zero gauge pressure; *T* is the temperature; *P* is the pressure; and *C* is the constant 0.0894.

(18) $${\hat{v}}_{0}\left(T\right)=\left\{\begin{array}{c} b_{1S}+b_{2S}\bar{T}\\ b_{1L}+b_{2L}\bar{T} \end{array}\right.\begin{array}{c} ,if\;T\leq T_{t}\\ ,if\;T>T_{t} \end{array}$$

(19) $$B=\left\{\begin{array}{c} b_{3S}\exp\left(-b_{4S}\bar{T}\right)\\ b_{3L}\exp\left(-b_{4L}\bar{T}\right) \end{array}\right.\begin{array}{c} ,if\;T\leq T_{t}\\ ,if\;T>T_{t} \end{array}$$

(20) $$v_{t}\left(T,P\right)=\left\{\begin{array}{c} b_{7}\exp\left(b_{8}\bar{T}-b_{9}P\right)\\ 0 \end{array}\right.\begin{array}{c} ,if\;T\leq T_{t}\\ ,if\;T>T_{t} \end{array}$$

(21) $$\bar{T}=T-b_{5}\;$$

(22) $$T_{t}=b_{5}+b_{6}P$$

where *v_t_* is the value for semi-crystalline resins only applies to temperatures below the transition temperature; T_t_ is used to characterize the abrupt viscosity change of the material around its transition temperature; 13 parameters (*b*_1_*_S_*, *b*_2_*_S_*, *b*_3*S*_, *b*_4*S*_, *b*_1*L*_, *b*_2*L*_, *b*_3*L*_, *b*_4*L*_, *b*_5_, *b*_6_, *b*_7_, *b*_8_, *b*_9_) are data-fitted coefficients. With only linear P-*v*-T transitions, b_7_, b_8_ and b_9_ are for amorphous materials.

### 3.2. Modeling of Analyzed Product

**Figure 5:**
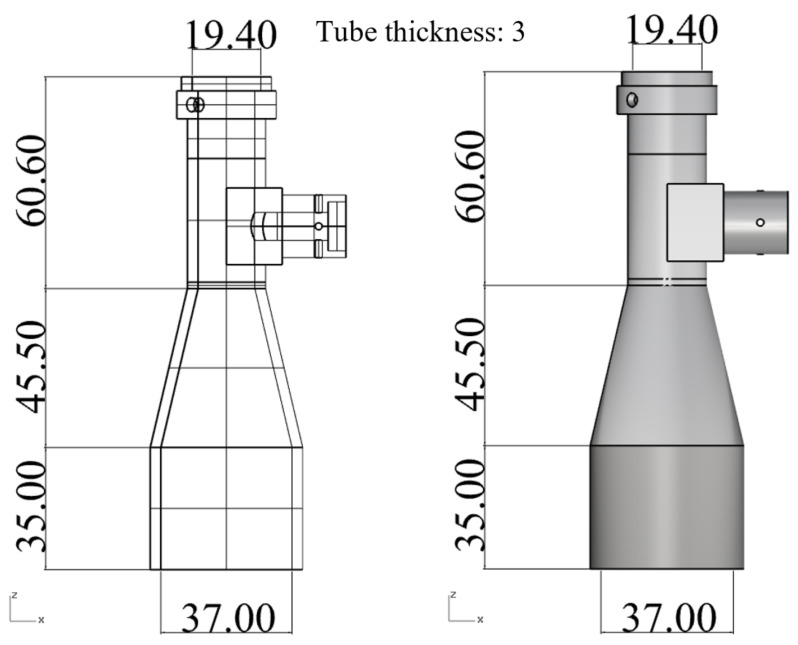
Designed geometry of telecentric lens (unit: mm).

The simulations considered a coaxial telecentric lens barrel with the dimensions and geometry shown in Figure 5.

**Figure 6:**
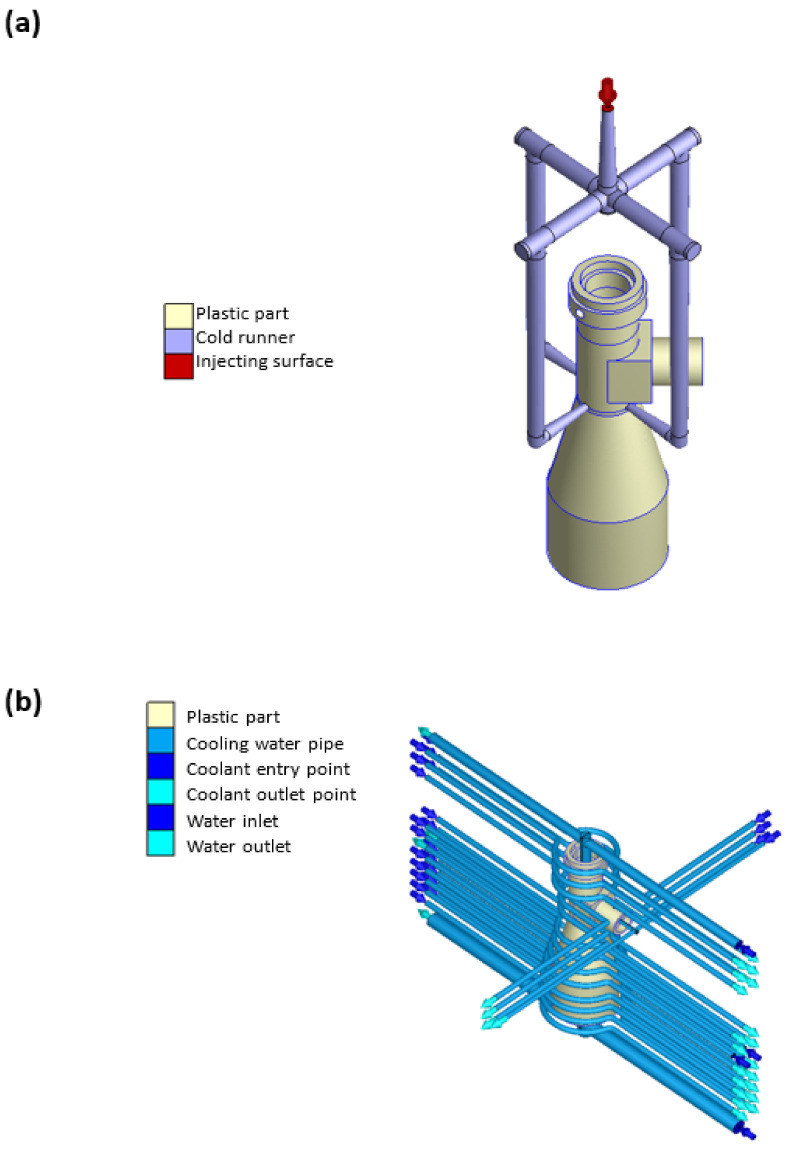
(**a**) Gating and (**b**) cooling systems for injection molding process.

Figure 6a,b show the gating and cooling system models used in the simulations. As shown in Figure 6a, the gating system was designed with four runners to accommodate the large component size and thin wall thickness (3 mm). Moreover, the cooling water runner system was numerically designed to fit snugly around the outside surface of the plastic barrel, and a baffle-type water runner was used to prevent internal heat accumulation (see Figure 6b).

### 3.3. Taguchi Design Method

**Figure 7:**
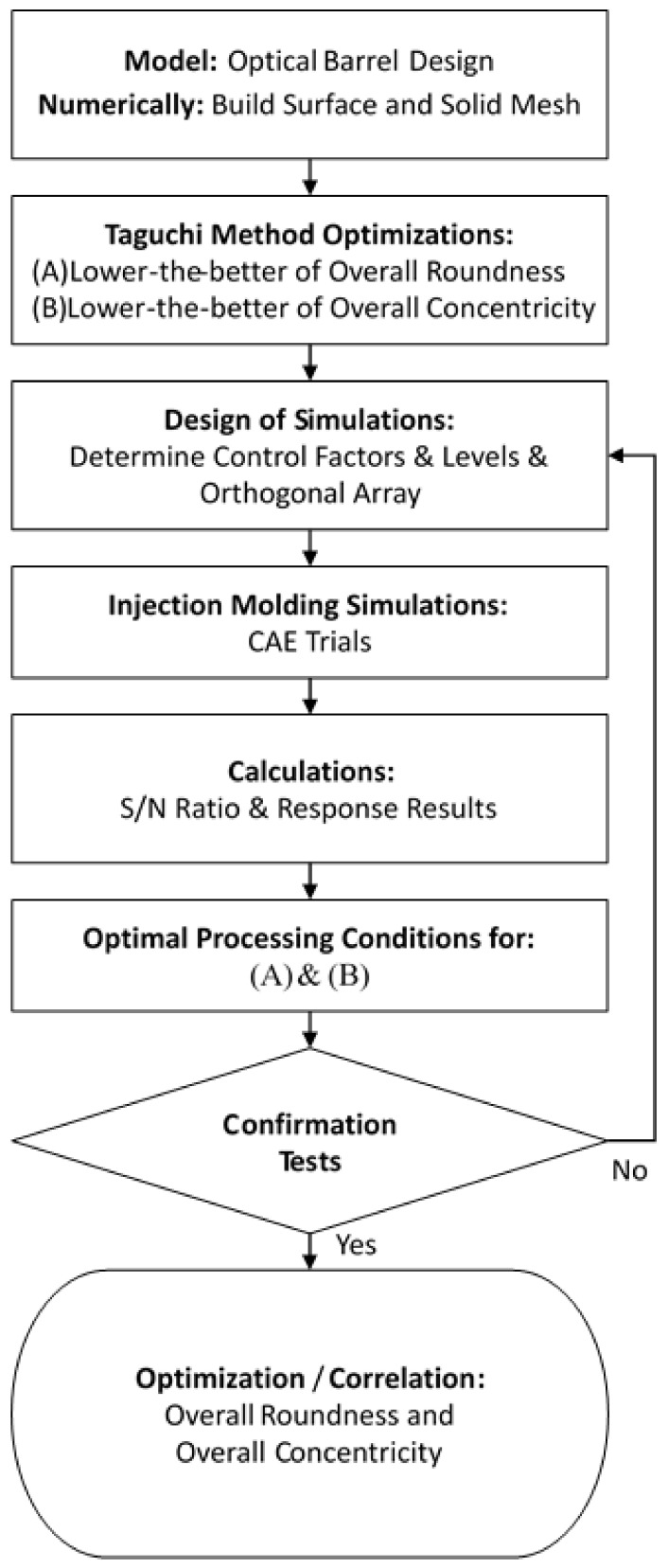
Flowchart showing main steps in Taguchi/CAE optimization process.

Figure 7 presents a flowchart of the hybrid Taguchi/CAE optimization process performed in the present study to identify the plastic injection molding processing parameters which minimize the overall roundness and overall concentricity of the optical barrel. As shown, the process commenced by constructing the numerical model described in the previous section and establishing the build surface and solid mesh. Having chosen suitable signal-to-noise (S/N) ratios for evaluating the quality of each simulation outcome, the Taguchi design processes were defined by establishing the control factors and level settings of interest. For some specific quality requirements such as deformation, warpage, shrinkage, weld line, air trap, roundness, concentricity, etc., each of these quality characteristics will have different influential processing factors. Therefore, in order to find the best combination of parameters, the Taguchi method is usually used to screen the most influential factors. This method is to utilize the statistical operation of the orthogonal array (OA) to find the optimal parameter combination. In Taguchi method, OA is a general partial factorial design. It is based on an orthogonal design matrix, allowing users to consider selected subsets of multi-factor combinations at multiple levels. Orthogonal arrays are balanced to ensure that all levels of all factors are considered equally in statistics. Other less influential parameters adopt the recommended values of polymer materials or injection molding machines. Generally speaking, the processing factors that have an influence on deformation are related to temperature and packing. After preliminary evaluation and calculation, five control factors were chosen, namely, (A) the injection pressure, (B) the packing pressure, (C) the melt temperature, (D) the mold temperature, and (E) the cooling time. As shown in Table 2, each of the five control factors was assigned four different level settings.

Thus, the Taguchi simulations were configured in an L_16_(4^5^) Orthogonal Array (OA), as shown in Table 3.

In the present study, the aim of the optimization process was to minimize the overall roundness and overall concentricity of the selected planes in the plastic barrel. Hence, in evaluating the quality of the solutions obtained from each simulation run in the OA, the smaller-the-better S/N ratio was adopted for both quality measures, i.e.,

(23) $$S/N=-10\log\left(\frac{1}{n}\overset{n}{\underset{i=1}{\sum }}y_{i}{}^{2}\right),$$

where $y_{i}$ is the roundness or concentricity and *n* is the number of measured points in the simulation trial.

### 3.4. Least Squares Circle Method for Evaluation of Roundness and Concentricity

**Figure 8:**
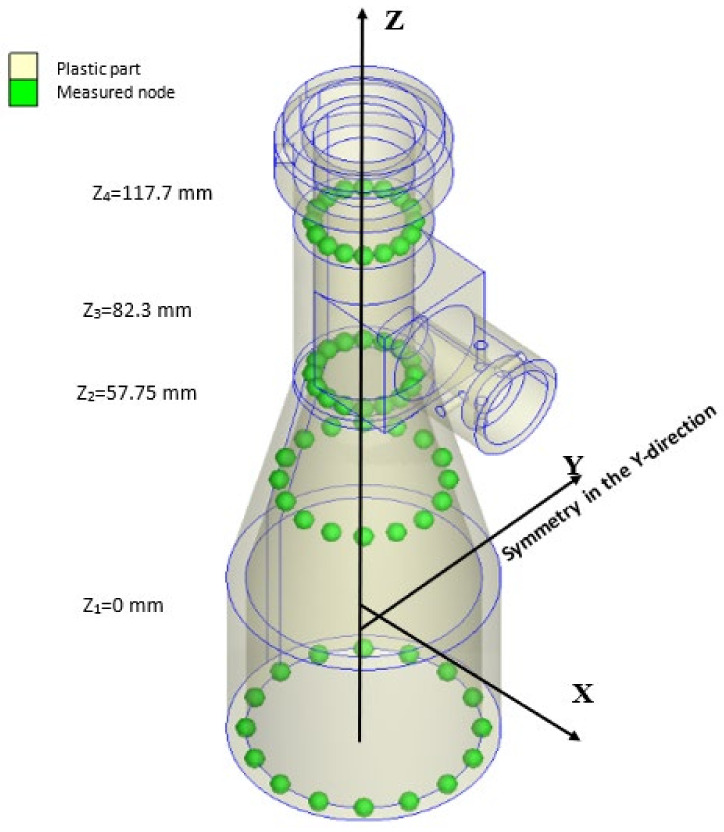
Measurement nodes used for roundness and concentricity evaluation at different Z-planes.

The roundness and concentricity computations were performed at four planes distributed along the barrel length, namely, Z = 0, Z = 57.75, Z = 82.3, and Z = 117.7 (mm), respectively, as shown in Figure 8. In determining the roundness using the LSC method (see Section 2.4), the center of the least squared error circle was determined using the function [42]:

(24) $$f\left(x,y\right)=\min\overset{n}{\underset{i=1}{\sum }}{\left[r\left(x,y\right)-R\right]}^{2}$$

where *r*(*x*, *y*) is the distance between the measured point and the known center of the circle, (*x*, *y*) are the coordinates of the measured point, *n* is the number of measured points, and *R* is the radius of the least square circle [43]. For each run in the OA array, the displacements of the measurement nodes (see Figure 8) were obtained and used to obtain the center point (Xc, Yc, Zc) and radius Rc (See Table 4) of the corresponding least square circle. As described in Section 2.4, the roundness is denoted by Δ*Zq*.

## 4. Results and Discussion

After Taguchi’s optimization calculation, including roundness, concentricity and correlation analysis, the relevant analysis results will be confirmed and discussed. Table 3 shows the average S/N values of the overall roundness and overall concentricity obtained in each of the 16 runs in the OA over the four measurement planes (Z_1_~Z_4_). The table also shows the S/N values obtained under the standard injection molding conditions for the injection machine and molding material (as prescribed by the manufacturer). Finally, the table shows the S/N values for the overall roundness and overall concentricity obtained under the optimal settings of the five control factors (see Section 4.1 below).

### 4.1. Factor Rank Analysis and Optimal Process Parameters

**Figure 9:**
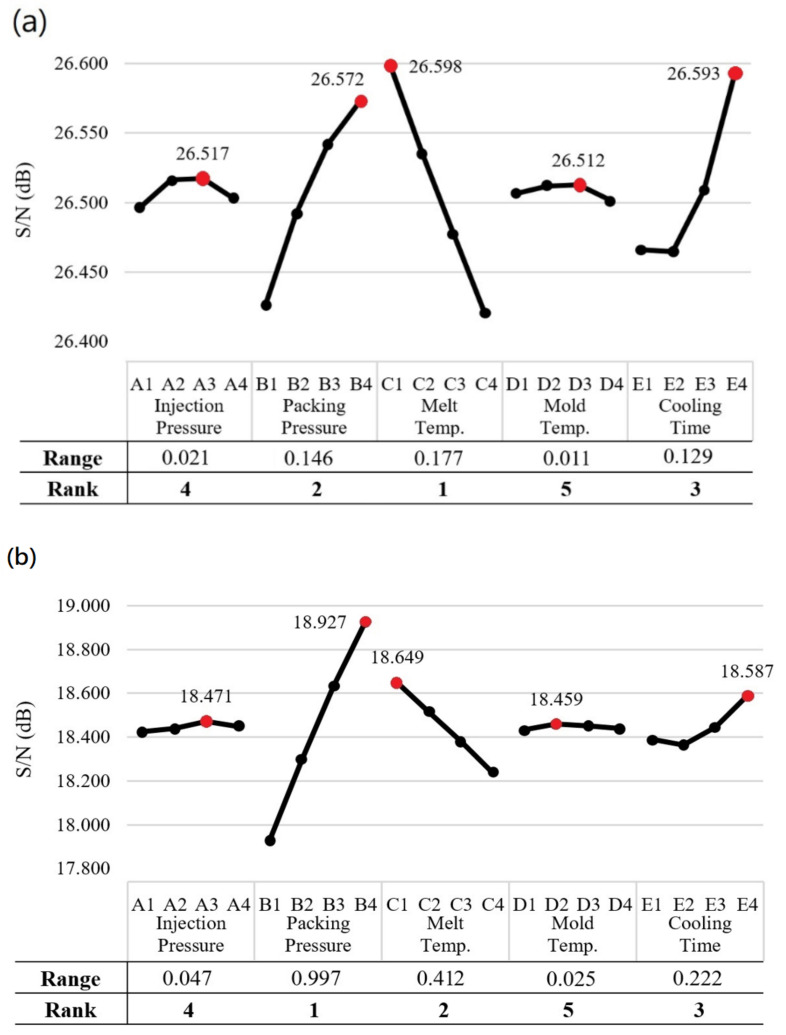
S/N response for (**a**) Overall roundness and (**b**) Overall concentricity.

Figure 9a,b show the Taguchi response graphs for the overall roundness and overall concentricity, respectively. Referring to Figure 9a, it is seen that the optimal overall roundness is obtained using factor level settings of A3, B4, C1, D3, and E4, i.e., an injection pressure of 220 MPa, a packing pressure of 240 MPa, a melt temperature of 275 °C, a mold temperature of 90 °C, and a cooling time of 17 s. Furthermore, the simulation results show that the overall roundness is dominated by the melt temperature (Rank 1), packing pressure (Rank 2), and cooling time (Rank 3) in sequence. By contrast, the injection pressure and mold temperature, with smaller S/N ranges of 0.020909 dB and 0.010688 dB, respectively, have only a relatively minor effect on the overall roundness. As shown in Table 3, the S/N value of the overall roundness under the optimal processing conditions (26.755 dB) is 0.283 dB higher than that of the barrel produced under the standard processing conditions (26.472 dB). Moreover, the S/N value is also higher than that produced in any of the simulation runs in the OA. In other words, the effectiveness of the optimized parameter design in minimizing the overall roundness of the molded plastic barrel is confirmed.

Figure 9b shows the Taguchi response graph for the overall concentricity of the molded barrel. It is seen that the optimal overall concentricity is again obtained using control factor level settings of A3, B4, C1, D3, and E4. The overall concentricity is determined mainly by the packing pressure (Rank 1), melt temperature (Rank 2), and cooling time (Rank 3). The injection pressure and mold temperature once again have only a minor effect on the overall concentricity. Referring to Table 3, it can be seen that the optimal processing conditions increase the S/N ratio (19.331 dB) by 1.053 dB compared with that obtained under the standard processing conditions (18.278 dB). In addition, the S/N ratio of the optimized design is higher than that obtained in any of the 16 runs of the OA. Thus, the effectiveness of the optimal processing conditions in improving the overall concentricity of the barrel is confirmed. Notably, the results presented in Table 3 show that the optimal values of the overall roundness and overall concentricity, respectively, are obtained using the same control factor level settings. In other words, the optimal design enables the simultaneous optimization of both the overall roundness and the overall concentricity.

The results presented in Figure 9a show that the packing pressure, melt temperature, and cooling time have similar S/N values, i.e., 0.146, 0.177, and 0.129 dB, respectively. In other words, all three factors exert a similar effect on the overall roundness of the molded barrel. However, the overall concentricity is dominated by a major factor, namely, the packing pressure (S/N = 0.997 dB) (see Figure 9b) and two moderate influence factors, namely, the melt temperature (S/N = 0.412 dB) and cooling time (S/N = 0.222 dB).

For the influence of packing pressure, when the mold cavity is completely filled with plastic melt, and the plastic melt will change from high temperature and high pressure to low temperature and low pressure. Due to changes in the temperature and pressure of the plastic melt, the final filled part may have obvious shrinkage in the mold cavity. Therefore, in order to overcome the shrinkage problem, the plastic melt in the runner will be continuously filled into the mold cavity when the filling stage is completed. This is called the packing stage of injection molding. In the packing stage, the inside of the mold cavity will reach the highest pressure, and the plastic melt will continue to solidify where it contacts the lower temperature mold wall. The packing process should continue until the injection gate is solidified. Generally speaking, increasing the packing pressure or extending the packing time will delay the curing time of the plastic melt, which will promote the dispersion of the pressure in the plastic part and reduce the volume shrinkage. Excessive packing pressure is likely to cause factors such as difficulty in demolding, high residual stress, burrs and flash. On the contrary, insufficient packing pressure will lead to larger volume shrinkage and voids and other defects.

The effects of the melt temperature and cooling time mainly affect the geometric deformation of the injected part. Deformation is the most important factor that simultaneously affects the optimization of both roundness and concentricity. Generally, the control factors affecting plastic deformation are temperature and cooling. The melt temperature can determine the difference between the surface compressive stress and the internal tensile stress of the injected part during the curing process. In addition, the cooling time is mainly because the semi-crystalline polymer needs sufficient time to crystallize during the cooling process to reduce the residual stress and the shrinkage.

### 4.2. Correlation between Overall Roundness and Overall Concentricity

**Figure 10:**
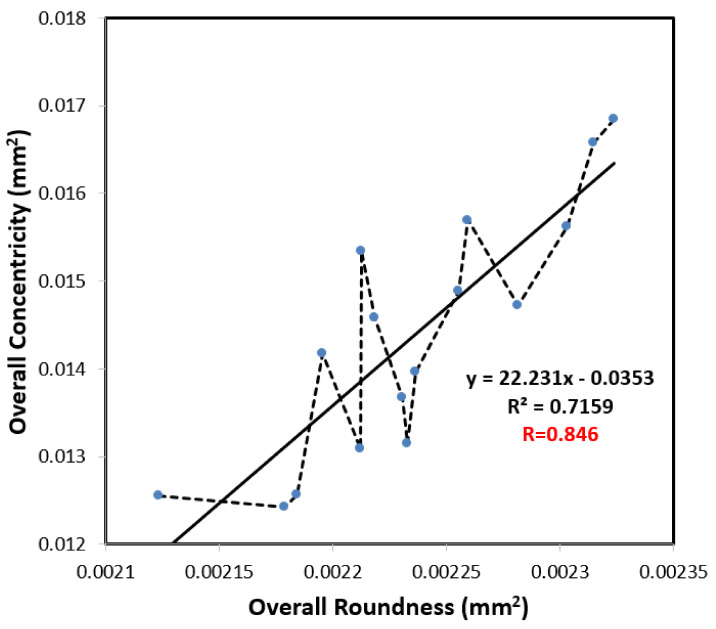
Analysis diagram of the correlation between overall roundness and overall concentricity.

Comparing the results presented in Figure 9a,b, it can be seen that the overall roundness and overall concentricity have identical trends in terms of their dependency on the level settings of each control factor. Furthermore, for both properties, the packing pressure, melt temperature and cooling time exert the greatest effect on the simulation outcome, while the injection pressure and mold temperature have only a minor effect. Figure 10 shows the results obtained when plotting the overall roundness values in Table 3 against the corresponding overall concentricity values. Applying a regression analysis technique to the simulation data, the correlation coefficient is determined to be R^2^ = 0.7159 (Or R = 0.846). Considering the general correlation evaluation, the correlation coefficient of the two variables is greater than 0.7, which can be regarded as highly correlated. In other words, the overall roundness and overall concentricity are positively related to one another, which explains why they respond in a similar manner to changes in the injection molding conditions and can be simultaneously optimized using the same control factor level settings.

### 4.3. Deformation and Shrinkage of Plastic Barrel

**Figure 11:**
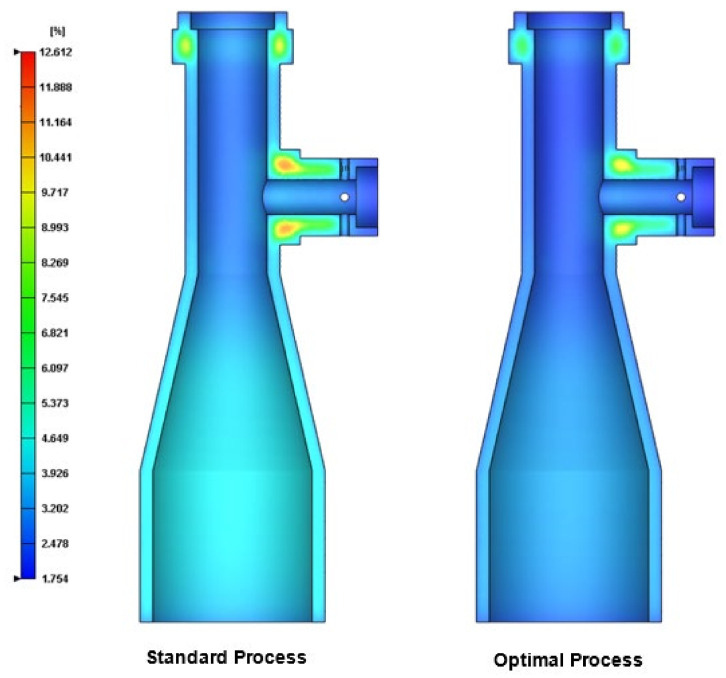
Cross-sectional shrinkage of final part processed using standard conditions (**left**) and optimal conditions (**right**). (Volume shrinkage: %).

**Figure 12:**
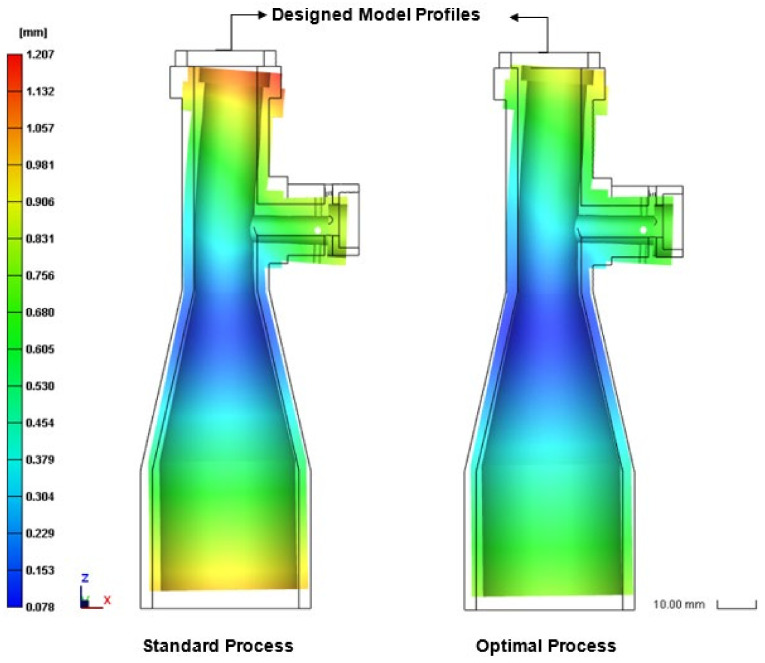
Side-view deformation of final part processed using standard conditions (**left**) and optimal conditions (**right**). (Displacement enlarged by factor of 10 for visualization purposes.).

**Figure 13:**
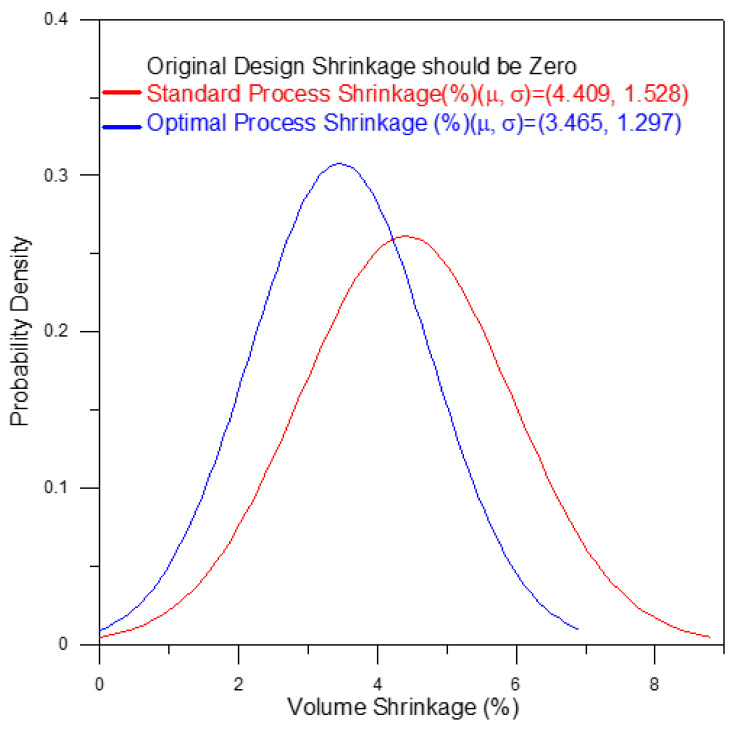
Shrinkage improvement of optimal process compared to standard process.

**Figure 14:**
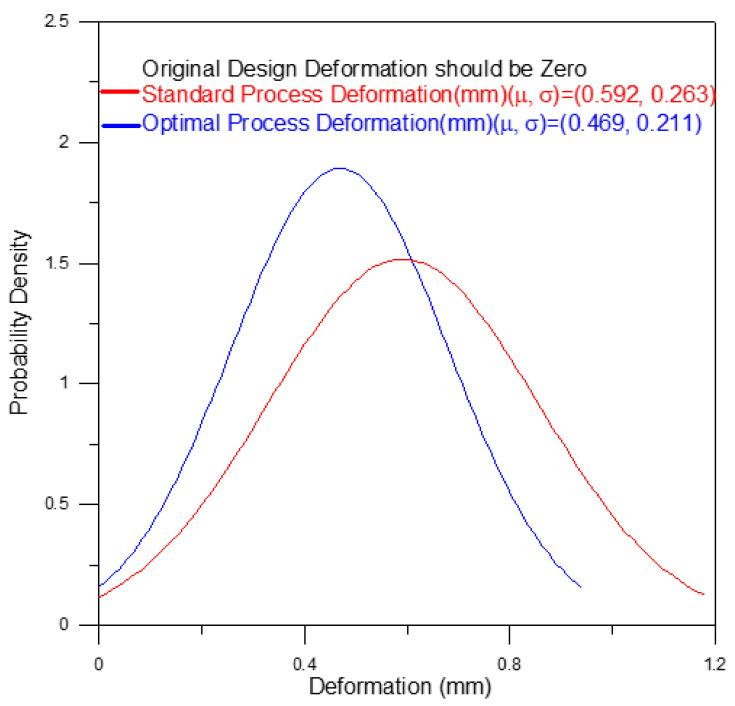
Deformation improvement of the optimal process compared to the standard process.

As shown in Figure 1, a holder structure is attached to the side of the lens barrel in order to support the barrel during use and maintain the coaxial condition of the light as it passes through the barrel. However, the addition of the holder induces a deformation of the molded barrel since the greater thickness of the holder structure relative to that of the barrel results in a corresponding reduction in the local cooling rate. Observing the left-hand schematics in Figure 11 and Figure 12, which show the barrel produced under the standard processing conditions, the local reduction in the cooling rate results in the formation of two regions of high-volume shrinkage due to the difference in cooling rates of the outer and inner regions of the holder structure, respectively. The greater volume shrinkage rate (Figure 11) then causes the narrower portion of the barrel to deform in the direction of the holder structure (Figure 12). However, as shown in the right-hand schematics in the two figures, the optimal processing conditions suppress the local volume shrinkage effect and reduce the barrel deformation accordingly. Figure 13 compares the overall volume shrinkage rates of the barrels produced using the standard and optimized processing conditions, respectively. (Note that in the ideal case, the volume shrinkage is equal to zero.) A detailed inspection shows that the optimal processing conditions reduce the overall average shrinkage rate and standard deviation from 4.409% to 3.465% and 1.528% to 1.297%, respectively. Similarly, Figure 14 compares the overall deformations of the barrels produced using the standard and optimized processing conditions and shows that the optimal processing conditions reduce the average deformations and standard deviation from 0.592 mm to 0.469 mm and from 0.263 mm to 0.211 mm, respectively.

### 4.4. Manufacturing and Processing Implications of Present Results

Table 4 shows the eccentric coordinates, least square circle radius, roundness, and concentricity values of the ideal telecentric barrel (original design) and barrels produced under the standard and optimal processing conditions, respectively.

Interestingly, the concentricity of the barrel produced under standard processing conditions at planes Z_2_ and Z_3_ (see Figure 8) is better than that of the barrel produced under the optimal process conditions at the same planes. It is speculated that this may be due to the holder. However, the overall roundness of the barrel produced using the optimal processing conditions is better than that of the barrel produced using the standard processing conditions at all of the measurement planes.

In general, the present results show that to improve the overall roundness and overall concentricity of the optical telecentric barrel, it is necessary to reduce the thermally induced residual stress to the greatest extent possible. The Taguchi optimization results suggest that this can best be achieved using an appropriate injection speed, increasing the packing pressure, extending the cooling time, reducing the product thickness difference, using an appropriate material temperature, reducing the mold temperature to improve the difference with the room temperature, and appropriately selecting the gate design.

## 5. Conclusions

In this study, the coaxial telecentric lens barrel was analyzed by considering the tolerances of the roundness and concentricity. Mold flow technology combined with the Taguchi design method were introduced to explore the roundness and concentricity arising from the shrinkage and deformation in the injection molding process. Five control factors and four levels of orthogonal array tables were selected for the Taguchi analysis. The results show that an appropriate selection of processing factors and levels can effectively optimize the roundness and concentricity of the lens barrel injection molding process. The simulation results support the following main conclusions.

- This study has successfully employed a hybrid Taguchi/CAE simulation approach to determine the optimal processing conditions which minimize the overall roundness and overall concentricity errors of an optical telecentric barrel produced using the plastic injection molding technique.
- The overall roundness and overall concentricity of the optical barrel are determined mainly by the packing pressure, melt temperature, and cooling time. Both properties can be improved by increasing the packing pressure, reducing the melt temperature, and extending the cooling time.
- The overall roundness and overall concentricity of the molded barrel are positively correlated with one another. Thus, an appropriate selection of the processing conditions optimizes both the overall roundness and the overall concentricity simultaneously.
- The holder structure added to the side of the lens barrel induces a local volume shrinkage effect which causes an axial deformation of the barrel. However, the optimal processing conditions reduce the overall volume shrinkage rate of the barrel from 4.409% (standard processing conditions) to 3.465% and reduce the overall deformations of the barrel from 0.592 mm (standard processing conditions) to 0.469mm. This paper successfully improves the overall roundness and overall concentricity in the vicinity of the holder structure accordingly.

## Figures and Tables

**Table 1:** Material properties of PA66 (TECHNYL A 216, Solvay Engineering Plastics; Source: Moldex3D material library).

Properties	Values	Unit
Density	1140	Kg/m^3^
Mold shrinkage	1.90	%
Water absorption (24 h at 23 °C)	1.30	%
Tensile modulus	3000	MPa
Tensile strength at break	55	MPa
Tensile strain at break	30	%
Flexural maximum stress	120	MPa
Melt temperature	263	°C
Heat conduction coefficient	0.25	W/(m•K)
Coefficient of linear thermal expansion (after demolding)(23 °C to 85 °C)	7	E-5/°C
Viscosity vs. shear rate under different temperature	See Figure 4a	
P-*v*-T	See Figure 4b	

**Table 2:** Control factors and level settings used in Taguchi simulations.

L_16_ (4^5^)	A Injection Pressure (MPa)	B Packing Pressure (MPa)	C Melt Temp. (^o^C)	D Mold Temp. (^o^C)	E Cooling Time (Sec)
**Level 1**	180	180	275	70	11
**Level 2**	200	200	280	80	13
**Level 3**	220	220	285	90	15
**Level 4**	240	240	290	100	17
**Standard Parameters**	200	200	280	80	13

**Table 3:** Taguchi analysis results for overall roundness and overall concentricity.

Trials	Processing Factors					Overall Roundness		Overall Concentricity	
	A Injection Pressure (MPa)	B Packing Pressure (MPa)	C Melt Temp. (°C)	D Mold Temp. (°C)	E Cooling Time (Sec)	$\overset{4}{\underset{i=1}{\sum }}{\left[{\left(\Delta Zq\right)}_{i}\right]}^{2}$/4 (mm^2^)	S/N (dB)	$\overset{4}{\underset{i=1}{\sum }}{\left(d_{i}\right)}^{2}/$4 (mm^2^)	S/N (dB)
**Standard** **Parameters**	**200**	**200**	**280**	**80**	**13**	**0.002253**	**26.472**	**0.01487**	**18.278**
**1**	180	180	275	70	11	0.002259	26.460	0.01569	18.043
**2**	180	200	280	80	13	0.002255	26.468	0.01488	18.274
**3**	180	220	285	90	15	0.002236	26.504	0.01397	18.549
**4**	180	240	290	100	17	0.002212	26.552	0.01309	18.832
**5**	200	180	280	90	17	0.002213	26.551	0.01534	18.141
**6**	200	200	275	100	15	0.002195	26.585	0.01417	18.486
**7**	200	220	290	70	13	0.002282	26.418	0.01472	18.321
**8**	200	240	285	80	11	0.002233	26.511	0.01314	18.813
**9**	220	180	285	100	13	0.002315	26.355	0.01658	17.804
**10**	220	200	290	90	11	0.002303	26.377	0.01562	18.062
**11**	220	220	275	80	17	0.002123	26.730	0.01256	19.012
**12**	220	240	280	70	15	0.002184	26.607	0.01257	19.007
**13**	240	180	290	80	15	0.002324	26.338	0.01684	17.736
**14**	240	200	285	70	17	0.002218	26.540	0.01458	18.362
**15**	240	220	280	100	11	0.002231	26.516	0.01367	18.641
**16**	240	240	275	90	13	0.002179	26.618	0.01242	19.058
**Roundness** **Optimized**	**220**	**240**	**275**	**90**	**17**	**0.002111**	**26.755**	-	-
**Concentricity** **Optimized**	**220**	**240**	**275**	**90**	**17**	-	-	**0.01167**	**19.331**

**Table 4:** The analyzed results of Roundness and Concentricity are on the four measured planes.

Unit: Mm	Measured Planes	Least Square Circles				Roundness (ΔZq)	Concentricity (d)
		Eccentric Coordinates			Radius		
		Xc	Yc	Zc	Rc		
**Original** **Model**	Z_1_ = 0	0	0	0	18.5	0	0
	Z_2_ = 57.75				13.25		
	Z_3_ = 82.3				8		
	Z_4_ = 117.7				8		
**Standard** **Processing**	Z_1_ = 0	1.740 × 10^−1^	−2.405 × 10^−4^	0.9380	18.25	0.005442	0.1740
	Z_2_ = 57.75	−6.099 × 10^−3^	−2.107 × 10^−4^	57.91	13.07	0.08299	0.006103
	Z_3_ = 82.3	−5.464 × 10^−2^	−1.300 × 10^−4^	82.13	7.896	0.03662	0.05464
	Z_4_ = 117.7	1.618 × 10^−1^	−6.092 × 10^−4^	117.0	7.916	0.02751	0.1618
**Optimal** **Processing**	Z_1_ = 0	1.528 × 10^−1^	−1.852 × 10^−4^	0.7284	18.31	0.004390	0.1528
	Z_2_ = 57.75	−1.698 × 10^−2^	−1.591 × 10^−4^	57.89	13.11	0.08240	0.01698
	Z_3_ = 82.3	−5.810 × 10^−2^	−9.810 × 10^−5^	82.17	7.918	0.03246	0.05810
	Z_4_ = 117.7	1.401 × 10^−1^	−5.558 × 10^−4^	117.2	7.934	0.02412	0.1401
